# Determinants of infant mortality in community of Gilgel Gibe Field Research Center, Southwest Ethiopia: a matched case control study

**DOI:** 10.1186/1471-2458-13-401

**Published:** 2013-04-27

**Authors:** Lamessa Dube, Mohammed Taha, Henok Asefa

**Affiliations:** 1 Department of Epidemiology, College of Public Health and Medical Sciences, Jimma University, Jimma, Southwest Ethiopia

## Abstract

**Background:**

Infant mortality accounts for almost 67 percent of under-five child mortality that occurs globally. An understanding of factors related to infant mortality is important to guide the development of focused and evidence-based health interventions to reduce infant deaths. But no community based studies have been conducted to identify determinants of infant mortality in Ethiopia for the past two decades. The purpose of this study is to identify determinants of infant mortality in community of Gilgel Gibe Field Research Center, Southwest Ethiopia.

**Methods:**

A community based matched case–control study was conducted. The study covered 133 infants who died during infancy between January 2010 and February 2011 in the study area. For each case, a control with approximately same date of birth and survived his/her first year of live and alive at time data collection was selected. Conditional logistic regression method was used to identify determinant factors of infant mortality using Epi-info 3.5.1 statistical software.

**Results:**

According to the final logistic regression model, not attending antenatal care follow-up [AOR=2.04, 95% CI:(1.04,4.02)], not using soap for hand washing before feeding child [AOR=2.50, 95% CI: (1.32,4.76)], negative perceived benefits of mother to modern treatment and prevention [AOR=2.76, 95% CI: (1.21,6.09)], small birth size [AOR=2.91, 95% CI: (1.01,8.46)] and high birth order with short birth interval [AOR=3.80, 95% CI: (1.20,11.98)] were found to be independent determinants of infant mortality.

**Conclusions:**

Antenatal care follow-up, hand washing habit with soap before feeding child, birth size, perceived benefits of mothers to modern treatment, birth order and preceding birth interval were determinants of infant mortality.

## Background

Infant mortality rate is defined as the risk for a live-born child to die before its first birthday. Infant mortality is known to be one of the most sensitive and commonly used indicators of the socioeconomic and health status of a country [1-3]. More than any other age group of a population, infant’s survival depends on the socioeconomic status of the family [3]. Reducing infant and child mortality by two third from 1990 to 2015 is one of the Millennium Development Goals (MDG) [4]. However, globally, there were 5.4 million infant deaths in 2010 which accounts for almost 67 percent of under-five child mortality. Out of this, about 43% of infant deaths occur in sub-Saharan Africa which is unacceptably high [5].

The conceptual framework for study of determinants of child survival includes proximate and distal (socioeconomic) determinants. This approach to the study of child survival is based on several premises [6]. Different studies reported that infant and under-five mortality is influenced by social–economic status [7-15], birth weight [16], mother’s age [10,17,18], sex of infant [10,19], breastfeeding [18,20-22], immunization [21], interval from previous delivery [10,21], birth order [10,22], parity [21,23], place of delivery [23] and etc.

The Ethiopian situation is similar with that of the Sub Saharan Africa which is characterized by high infant mortality rate; it also ranks 6^th^ in the world by total number of death of infants [5]. Infant and child mortality in Ethiopia had shown a continuous decline since 1960 onwards with a more pronounced reduction in the recent decades. The trend of infant mortality rates has been about 200 per 1000 live births in 1960, 153 per 1000 live births in 1970, 110 per 1000 live births in 1984, 97 per 1000 live births in 2000 and 77 per 1000 live births in 2005, 59 per 1000 live birth in 2011. This means that infant mortality declined by 20.6% and 23% between 2000 to 2005 and 2006 to 2011 respectively [24,25]. This decline may be attributed to expansion of Health Extension program, high coverage of immunization, community based intervention, IMNCI, and rapid expansion of health facilities. According to the 2011 Ethiopian Demographic Health Survey (EDHS) there were 59 deaths per 1000 live births. But it contributes to 67% of the under-five children mortality [25]. Regarding the study area, recorded data from Gilgel Gibe Field research center showed 65/1000 infant mortality rate which is higher than the national figure of EDHS 2011. Even though the trend of infant mortality declining in Ethiopia, it is still intolerably high.

Cohort study was conducted on determinants of infant mortality in 1992–94 in Southwest Ethiopia including Gilgel Gibe field research center, had reported that birth weight, sex of infants, types of birth, marital status, education, family size, Antenatal care follow-up and soap use were determinants of infant mortality [11]. Since then studies were not done in this area on specified title even though infant mortality in the area is above the national average.

The Federal Ministry of Health of Ethiopia has developed national strategy for child survival by 2005 with the overall objective to reduce under-five mortality by 52% from 2004 baseline. To realize this goal, the document recommended the higher institutions to take responsibility in the development of research proposals, with special emphasis on community-based research, which will ultimately be relevant for child survival [26].

Therefore, the purpose of the study is to identify and see any change in determinants of infant mortality that would help in planning and implementing interventions to reduce infant deaths.

## Methods

### Study setting, design and sampling

A matched case–control study was conducted from March 1 to 30, 2012 at Gilgel Gibe Field Research Center. The Gilgel Gibe Field Research Center [GGFRC] setting was identified by Jimma University as a field research center and setting for Community Based Education. There are eight rural and two urban kebeles in the area. In 2011, the total population of the study area was about 50,000 within 10,500 households. According to GGFRC record 2,120 infants were born alive in the study area from January 1, 2010 to February 29, 2011. The study population included all cases identified and their controls registered in the research center database. Cases were all infants who were born alive from January 1, 2010 to February 29, 2011 and died before celebrating their first birth day. For each case, a control with date of birth within two days and alive at time of data collection was selected.

The number of cases and controls required for the study were determined using sample size formula for matched case–control studies [27].

$$m=\frac{{\left[Z\alpha \left(\psi +1\right)+2Z\beta \sqrt{\psi }\right]}^{2}}{{\left(\psi -1\right)}^{2}}\;n=\frac{m}{\mathrm{pd}}$$

Where

*m* = numbers of discordant pairs

*n* = sample size in pairs

*ψ* = = Odd ratio calculated by [*p*_*1*_*(1-p*_*2*_)]/[*p*_*2*_*(1-p*_*1*_*)],* where p_1_ and p_2_ are the exposure rate of the case and control groups.

*P*_*d*_ = the proportion of discordant pairs, = *p*_*2*_*q*_*1*_*+p*_*1*_*q*_*2*_

&Zeta;*α* = value of standard normal distribution corresponding to a significance level of alpha

&Zeta;*β* = value of standard normal distribution corresponding to the desired level of power of the test

As estimated from study done in Iran, proportion of mother’s age at first birth less than 18 years (main exposure variable) among the cases and controls are 47.8% and 33.7% respectively (28). At 5% level of significance, power of 90% and non-response rate of 5%, the number of cases and controls required were 266 (133 cases and 133 controls).

### Measurements

Data were collected from mothers via face to face interview using structured questionnaire adapted from another study [21]. Socio-economic and proximate factors of infant mortality were addressed. In the proximate determinants, the following were included: reproductive factors, environmental factor, hygienic factor, nutritional factor, child factor and behavioral factors which included practice, knowledge and perception. The questionnaire was initially adapted in English and translated into local language. The instrument was pre-tested in 5% of sample size. The data were collected by five trained and experienced GGFRC fieldworkers who were familiar with the area and speak the local language. Field questionnaires were checked daily for its completeness by supervisors.

Wealth score index was measured based on six dimensions: income greater than 500 Ethiopian Birr per month, presence of radio or television, availability of latrine, safe water supply and roof of house made of corrugated iron sheets were given score one and other categories were given score 0. The sum was computed and respondents who scored above the mean were labeled as satisfied basic need and otherwise labeled as poor. Eight knowledge questions on common cause of infant morbidity and mortality and its prevention were presented and correct answer scored 1 and incorrect answer score 0. The sum was computed and those who got above the mean were labeled as having ‘good knowledge’ otherwise poor knowledge.

Perceived severity of common diseases, perceived susceptibility of child to common diseases, perceived benefits and barriers of common prevention methods were assessed using Likert Scale Method (1. strongly agree 2. agree 3. neutral 4. disagree 5. strongly disagree). Mean scores for each construct was computed and dichotomized into positive and negative. If a respondent scores below the mean she was labeled as having positive perceived severity, susceptibility, benefits and barriers, otherwise negative. Four questions related to practice were asked. These questions were related to breast milk, complementary feeding, Oral Rehydration Salt (ORS), use of Insecticide Treated bed Nets (ITN) and place of treatments when a child is sick. Correct answer scored 1 and incorrect answer scored 0. The sum was computed and those scoring above the mean were labeled as having good practice otherwise poor. Birth size is size of child at birth, which is considered as a proxy of birth weight, based on perception of the mother.

### Analysis

Data were entered, processed and analyzed by EPI INFO software version 3.5.1. Data analysis was started by describing each variables involved. Bivariate conditional logistic regression models were fitted for each explanatory variable separately to identify those associated with infant mortality. Variables with p-value less than 0.20 in the bivariate analysis were considered as candidate to be entered in the multivariate model. The using back ward elimination method the final model was fitted. Log likelihood ratio test was used to assess the goodness of fitness of the final model.

The ethical clearance was obtained from Jimma University. Written informed consent was also obtained from each respondent.

## Results

A total of 254 infants’ mothers were interviewed, having the response rate of 95.5%. All of the infants were singleton birth. Fifty-eight percent case infants were males. Almost 96% of the mother’s of both cases and controls were Oromo by their ethnicity. Ninety percents of mother’s of cases were illiterate. The mean (±SD) age of (mothers was 29.9(5.7) (Table 1).

**Table 1:** Socio-demographic characteristics of the study participants and their mothers, Gilgel Gibe Field Research Center, March 2012

**Variable**	**Case (n=127)**	**Control (127)**
	**Number (%)**	**Number (%)**
**Ethnicity**		
Oromo	122(96.1)	123(96.9)
Others	5(3.9)	4(3.1)
**Religion**		
Muslim	124(97.6)	123(96.9)
Christian	3(2.4)	4(3.1)
**Education status of mother**		
Illiterate	115(90.6)	102(80.3)
Literate	12(9.4)	25(19.7)
**Occupation of mother**		
Housewife	92(72.4)	95(74.8)
Farmer	31(24.4)	30(23.6)
Merchant	4(3.1)	2(0.8)
**Age of mothers (years)**		
<20	5(3.9)	3(2.4)
20-34	85(66.9)	98(77.2)
≥35	37(29.2)	26(20.4)
**Marital status**		
Married	121(95.3)	126(99.2)
Widowed	6(4.7)	1(0.8)
**Sex of infants**		
Male	74(58.3)	73(57.5)
Female	53(41.7)	54(42.5)

Among 127 deaths included in the present study, 24 (18.9%), 29 (22.8%) and 74(58.3%) were died during the first 24 hours after birth, after 24hours to 28 days and during post-neonatal (29 to 364 days) respectively.

The minimum and maximum household’s monthly income was 100 and 4500birr for cases and 100 and 6000birr for controls. The median monthly income was 350 birr for cases and 450 for controls. The mean (± SD) age of weaning by months among case and control were 4.5 (± 1.90) and 5.3 (± 1.40) respectively.

During bivariate analysis, educational status of mother, wealth index, family size, lack of radio, ANC follow up, birth order with birth interval, birth size, immunization status of child, soap use for hand washing before feeding child, availability of latrine, type of roof, availability of windows, perceived benefits of mothers on modern treatments and prevention and good mother’s practice concerning breastfeeding, ORS use during diarrhea, ITN utilization and taking ill infants to health facility were significantly associated with infant mortality. However, other variables like educational status of father, occupation of mother, age of mother at childbirth, age at first marriage, age at first birth and place of delivery were not significantly associated (Table 2).

**Table 2:** Univariate analysis for the association between the selected explanatory factors and infant mortality, Gilgel Gibe Field Research Center, March, 2012

**Variable**	**Cases**	**Controls**	**crude OR**	**95% CI**	**P-value**
	**(n=127)**	**(n=127)**			
	**Number (%)**	**Number (%)**			
***Socio-economic factors***					
Mother’s educational levels					
Literate	12(9.4)	25(19.7)	1		
Illiterate	115(90.6)	102(80.3)	2.62	1.16-5.92	0.02*
Residence					
Urban	13(10.2)	26(20.5)	1		
Rural	114(89.8)	101(79.5)	2.86	1.21-6.75	0.017*
Father’s Educational levels					
Literate	31(24.4)	39(30.7)	1		
Illiterate	96(75.6)	88(69.3)	1.4	0.79-2.22	0.2
Monthly Income					
Poor	90(70.9)	67(52.8)	2.35	1.33-4.14	0.003*
Satisfied basic need	57(29.1)	60(47.2)	1		
Occupation of mother					
Housewife	92(72.4)	95(74.8)			
Farmer/Merchant	35(27.6)	32(25.2)	1.17	0.62-2.19	0.631
Family size					
≤ 5	43(33.9)	65(51.2)	1		
>5	84(66.1)	62(48.8)	2.10	1.23- 3.58	0.005*
Radio possession					
Yes	40(31.5)	62(48.8)	1		
No	87(68.5)	65(51.2)	2.38	1.32- 4.26	0.02*
Age of mother at birth the child					
<20	10(7.9)	9(7.1)	1.18	0.45-3.06	0.741
20-34	97(76.4)	107(84.3)	1		
≥35	20(15.7)	11(8.7)	2.14	0.92-4.96	0.077
***Reproductive health and related factors***					
Age at marriage					
< 18	78(61.4)	69(54.3)	1		
≥ 18	49(38.6)	58(45.7)	1.32	0.80-2.16	0.267
Age at first birth					
< 20	93(73.2)	93(73.2)	1		
≥ 20	34(26.8)	34(34.8)	1	0.58-1.70	1.0
Antenatal care follow-up					
Yes	32(25.2)	58(45.7)	1		
No	95(74.8)	69(54.3)	2.62	1.47-4.66	0.001£
Place of delivery					
Home	121(95.0)	120(94.5)	1		
Health Institution	6(5.0)	7(5.5)	0.86	0.28-2.55	0.782
***Child and nutrition related factors***					
Sex of the infant					
Male	74(58.3)	73(57.5)	1		
Female	53(41.7)	54(42.5)	0.9	0.57-1.62	0.894
Immunization**					
Yes	23(32.7)	51(71.8)	1		
No	48(67.8)	20(28.2)	4.11	1.98 -8.52	0.001£
Size of child at birth					
Average and above	106(83.5)	117(92.1)	1		
Small	21(16.5)	10(7.9)	2.57	1.07-6.16	0.028
Birth order and interval					
Order 2–4 & Interval ≥2 yrs	33(26.0)	62(48.8)	1		
Order =1	10(7.9)	10(7.9)	1.55	0.57-4.18	0.382
Order 2–4 & Interval < 2 yrs	10(7.9)	4(3.1)	5.28	1.48-18.81	0.010*
Order ≥ 5 & Interval ≥2 yrs	57(44.9)	43(33.9)	2.49	1.35-4.60	0.002*
Order ≥ 5 & Interval < 2 yrs	17(13.4)	8(6.3)	4.23	1.49-11.95	0.006*
Complementary feeding**					
At 6 months	25(43.9)	85(66.9)	1		
Less than 6 months	32(56.1)	42(33.1)	6.67	1.98-22.44	0.001£
***Household environmental factors and hygiene***					
Latrine Availability					
Available	102(80.3)	116(91.3)	1		
Not available	25(19.7)	11(8.7)	2.56	1.18-5.52	0.013*
Water source					
Safe	60(47.3)	72(58.7)	1		
unsafe	67(52.7)	55(41.3)	1.57	0.91-2.72	0.105
Roof type					
Thatched	100(78.7)	83(65.4)	1		
CIS	27(21.3)	44(34.6)	0.49	0.27-0.88	0.018*
Availability of window					
Available	25(19.7)	44(34.6)	1		
Not available	102(80.3)	83(65.4)	2.19	1.21-3.95	0.010*
Number of rooms of house					
≥2	97(76.4)	102(80.3)	1		
< 2	30(23.6)	25(19.7)	1.46	0.68-3.13	0.335
Soap use for hand washing^**@**^					
Yes	47(37.0)	74(58.3)	1		
No	80(63.0)	53(41.7)	2.58	1.47-4.52	0.001*
***Behavioral Factors***					
Knowledge					
Satisfactory	46(36.2)	52(59.1)	1		
Not satisfactory	81(63.8)	75(40.9)	1.26	0.73-2.17	0.406
Perceived Susceptibility					
Positive	72(56.7)	79(62.2)	1		
Negative	55(43.3)	48(37.8)	1.28	0.75-2.15	0.353
Perceived Severity					
Positive	73(57.5)	80(63.0)	1		
Negative	54(42.5)	47(37.0)	1.31	0.75-2.16	0.327
Perceived Benefits					
Positive	64(50.4)	83(65.4)	1		
Negative	63(49.6)	44(34.6)	2.46	1.29-4.69	0.004£
Perceived Barriers					
Positive	50(39.4)	62(48.8)	1		
Negative	77(60.6)	65(51.2)	1.55	0.90-2.64	0.108
Practice					
Good	45(36.0)	81(63.28)	1		
Poor	80(64.0)	46(36.2)	3.83	2.03-7.24	0.001£

^*^P <0.05 ^**^ the total may not be equal to254, ^£^P<0.001 CIS- Corrugated Iron sheet.

^**@**^ Soap use for hand washing before feeding child.

Finally, ANC follow up during pregnancy, size of child at birth, mother’s perceived benefits on modern treatment to common diseases, mothers hand washing with soap, birth order and birth interval were independent determinants of infant mortality (Table 3).

**Table 3:** Factors independently associated with infant mortality, Gilgel Gibe Field Research Center, March, 2012

***Variables***	***AOR(95%CI)***	***P-value***
ANC follow up		
Yes		
No	2.04(1.04-4.02)	0.03*
Size of child at birth		
Average/ large size		
Small size	2.91(1.01-8.47)	0.04*
Birth order and interval		
Order 2–4 & Interval ≥2 yrs	1	
Order =1	2.13(0.67-6.77)	0.19
Order 2–4 & Interval < 2 yrs	4.14(1.10-15.55)	0.035*
Order ≥ 5 & Interval ≥2 yrs	2.59(1.30-5.19)	0.007*
Order ≥ 5 & Interval < 2 yrs	3.80(1.20-11.98)	0.012*
Soap use for hand washing^**@**^		
Yes	1	
No	2.50(1.32-4.76)	0.005*
Perceived benefit		
Positive	1	
Negative	2.75(1.01-6.09)	0.012*

*P<0.05 ^**@**^ Soap use for hand washing before feeding child**.**

The study showed that, infants whose mothers had no ANC follow up were more likely to die than those whose mothers had at least one follow up [AOR= 2.04, 95% CI: (1.04 -4.02)]. The risk of infant mortality among small size babies at birth were higher compared to those average and larger sized [AOR=2.91, 95% CI: (1.01-8.47)]. Among infants whose birth order were two through four, the risk of dying were higher for those with preceding birth interval less than two years compared to those with more than or equal to two years of spacing [AOR= 4.14, 95% CI: (1.10 - 15.55)]. Compared to those who were born after more than or equal to two years of spacing and their birth order second to fourth, the risk of dying were 2.7 times higher among infants who were born after spacing of greater than or equal two years from the preceding birth and were fifth birth order or above [AOR=2.70, 95% CI: (1.39 - 5.19)]. Hand washing with soap before feeding child was found to be significant in determining infant mortality. Infants whose mothers did not use soap for hand washing were more likely to die than whose mothers used soap [AOR= 2.50, 95% CI: (1.32 -4.76)]. Of behavioral factors, perceived benefit of modern treatment of common diseases was found to be significant in determining infant mortality. The risks of dying among infants whose mothers had negative perceptions on modern treatment were higher compared to infants whose mothers had positive perceived benefits [AOR= 2.75, 95% CI: (1.01-6.09)].

## Discussion

This study intended to identify the determinants of infant mortality. The study use analytic matched case control design. However, it might have some limitation such as: first, recall bias on risk factors. Second, survivals of infants are related to the past whereas available measures of household income and mother knowledge are current measures. Therefore, in this study, current income is a proxy for the past year income and current knowledge is a proxy for the past year knowledge. Third, birth weight of infant was estimated by birth size as reported by mother, which is based on mother’s perception. Fourth, some variables like postnatal care, access to health care, maternal tetanus toxoid immunization status, nutritional status of mother during pregnancy, health status of mother during pregnancy and nutritional status of child were not explicitly assessed by this study. Therefore, any reader of this manuscript should take in to account the above limitations.

The analysis of breast feeding was restricted to infants older than 7 days to avoid including neonatal deaths that were not likely related to infant feeding mode; to avoid reverse causation. It was hypothesized that the time when colostrums would start to provide protection to the infant is 7 days after birth [14]. In this study the impact of breastfeeding was not analyzed because it was universal. Immunization was considered for infants lived greater than 42 days. In this study parity and birth order were perfectly similar. For this reason only birth order was used in the analysis.

In this study, maternal education is found to be not associated with infant mortality. Similar studies done in Kenya, Pakistan and Iran also showed that no significant association between infant mortality and maternal education [10,18,28]. However, other studies reported that there were association between mother’s education and infant mortality [11,29]. In this study, it could be because of majority (85.4%) of mothers involved were illiterate.

Concerning maternal age at birth of the child, even though, it is in same direction with previous studies [10,12,18,22,30], it was not significantly associated in this particular study. This could be because of similarity of mother’s age distribution among both comparison groups.

Previous studies had reported significant association between fertility variable (short preceding birth interval and birth order) and infant mortality [10,15,18,28-30]. Similarly in this study, the combination of a higher birth order and a shorter interval had a higher risk of dying than lower rank with a longer birth interval. Those infants whose mothers had many children were more likely to die compared to infant whose mothers had given few births. This could be related to lower maternal nutrition status due to repeated pregnancy, resource competition among siblings, lack of adequate care and attention experienced by high-ranked infants. It could also be because as family size grows the parental resources might not growth as well leading to difficulty to maintain the same level of nutrition for a larger number of children [10,15,18,30].

ANC follow-up was associated with infant mortality. Those infants whose mothers had no ANC follow-up during pregnancy had twice more likely to die than whose mothers had at least one follow-up. Similarly, studies done in southwest Ethiopia, Brazil and India showed that, there is association between ANC follow-up and infant mortality [11,16,22]. This could be because the mothers who attended antenatal care during pregnancy are more likely to utilize existing health services and they can properly consume such services when wanted for their child [22]. Also Antenatal care protect early infant mortality through improving mothers nutritional status during pregnancy, Tetanus Toxoid Immunization, giving care to mothers health events, either chronic disease or acute disease, during pregnancy and reducing prevalence of low birth weight by improving nutritional status of mother during pregnancy [16].

Small birth size was one of the determinants of infant mortality. This had been reported by previous studies done Ethiopia, Brazil and Indonesia [11,16,31]. Birth size reflects the quality of care given to the mother, nutritional status of the mother and health status of mother during pregnancy [16].

Immunization practice is directly related with health status of infant. Associations had been previously reported between immunization and the risks of infant mortality [14,15,17,23]. Similarly, in this study the bivariate analysis showed that immunization is associated to infant mortality. However, this analysis focuses on those infants who survived at least 42 days of age.

In this study hand washing habit with soap before feeding child was significantly associated with infant mortality. Similar finding was reported by other study done previously in Southwest Ethiopia [11].

Among behavioral factors, there was a significant association between negative perception on benefits of some modern treatment and infant mortality. This result is in line with study done previously on determinants of under-five mortality [14]. This might be because mothers who had positive perceived benefits were more likely to take or to use modern treatment and prevention methods. Mothers who had negative perception on the benefit of some treatment might seek help from traditional healer which might not be helpful for the survival of the child [14].

## Conclusions

This study identified ANC follow up, birth size, hand washing habit of mothers with soap before preparation of food and feeding, perceived benefits of modern treatment, birth order and birth interval as a determinants of infant mortality. Therefore promoting antenatal care follow up for all pregnant women, reducing the higher birth order and prolonging short birth interval though family planning will have the substantial effect in reducing the risk of infant mortality. Secondly promotion of hand washing with soap while preparing food and infant feeding is also recommended. Thus, national public health intervention at primary health care level (e.g. Health extension workers) to improve infant survival should focus on these determinants to reduce infant mortality. Additionally further study including the above missed variables is also recommended.

## Competing interests

The authors declare that they have no competing interests.

## Authors’ contributions

LD involved from the inception to design, acquisition of data, analysis and interpretation, drafting the manuscript, MT involved in the inception to design, analysis and interpretation and revise critically the manuscript. HA involved in the inception to design, analysis and interpretation and revise and edit the manuscript for the final submission. All authors read and approved the final manuscript.

## Pre-publication history

The pre-publication history for this paper can be accessed here:

http://www.biomedcentral.com/1471-2458/13/401/prepub
